# A complementary food supplement from local food ingredients to enhance iron intake among children aged 6–59 months in Benin

**DOI:** 10.1002/fsn3.2358

**Published:** 2021-06-10

**Authors:** Marius Affonfere, Flora Josiane Chadare, Finagnon Toyi Kévin Fassinou, Elise F. Talsma, Anita R. Linnemann, Paulin Azokpota

**Affiliations:** ^1^ Laboratoire de Sciences et Technologie des Aliments et Bioressources et de Nutrition Humaine Ecole des Sciences et Techniques de Conservation et de Transformation des Produits Agricoles, Centre Universitaire de Sakété Université Nationale d’Agriculture Sakété République du Bénin; ^2^ Laboratoire de Sciences des Aliments Faculté des Sciences Agronomiques Université d’Abomey‐Calavi Jéricho, Cotonou République du Bénin; ^3^ Division of Human Nutrition and Health Wageningen University and Research Wageningen The Netherlands; ^4^ Food Quality and Design Wageningen University and Research Wageningen The Netherlands

**Keywords:** anemia, food‐to‐food fortification, food supplementation, minerals

## Abstract

Nutritious complementary feeding is often not affordable in Benin, and iron deficiency exists. This research aimed at formulating an affordable and sensory acceptable complementary food supplement using local food ingredients to increase iron intake among children aged 6–59 months in Benin. The complementary food supplement was formulated to ensure that 10 g would cover 25% of the estimated average requirements for iron for children aged 6 to 12 months. *Adansonia digitata* fruit pulp, *Moringa oleifera* leaf powder, and *Cochlospermum tinctorium* root powder were used to compose the complementary food supplement, which was mixed with maize and sorghum ogi porridges before being presented to the mothers and children for the acceptability test. The mineral contents of *Adansonia digitata* fruit pulp in mg/100 g dw were 9.9 ± 0.1 for iron and 0.9 ± 0.1 for zinc. The iron and zinc contents of *Moringa oleifera* leaf powder and *Cochlospermum tinctorium* root powder in mg/100 g dw were 34.1 ± 2.2 and 26.8 ± 2.7 and 9 ± 0.0 and 0.9 ± 0.0, respectively. The complementary food supplement contained, in mg/100 g dw, 17.4** ± **1.1 of iron and 1.2 ± 0.1 of zinc. The maize and sorghum ogi porridges enriched with the complementary food supplement at substitution rates of 15% and 16% (in dry weight), respectively, were acceptable to 85% of children for sorghum ogi porridge and 87% for maize ogi porridge. The present study demonstrated the potential of local food ingredients in the formulation of an iron‐rich and acceptable complementary food supplement for children aged 6–59 months in Benin.

## Graphical abstract

This research aimed at formulating an affordable and sensory acceptable complementary food supplement using local food ingredients to enhance iron intake among children aged 6–59 months in Benin. The complementary food supplement was formulated to ensure that 10 g would cover 25% of the estimated average requirements for iron for children aged 6 to 12 months. The designed complementary food supplement contained 17.4 ± 1.1 mg/100 g dw of iron, 1.2 ± 0.1 mg/100 g dw of zinc, and 830.0 ± 0.2 mg/100 g dw of calcium.

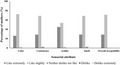

## 1 INTRODUCTION

Malnutrition, especially micronutrient deficiencies (MNDs), among infants is an important public health problem. Worldwide, MNDs affect an estimated 2 billion people, in particular women and children under 5 years (Bailey et al., 2015; Kassebaum et al., 2014; Saini et al., 2014), which can be related to the low nutrient density of the complementary foods, inadequate complementary feeding practices, child care, and sanitation (Akombi et al., 2017; Issaka et al., 2015). Most complementary foods consumed by infants in many parts of the world are reported to be deficient in essential micronutrients (Adeoti & Osundahunsi, 2017). These complementary foods are usually based on products derived from cereals (Arise et al., 2014; Muhimbula et al., 2011; Van der Merwe et al., 2019) and sometimes fortified with nutrient‐dense plant species (Abioye & Aka, 2015; Adejuyitan et al., 2012). Sorghum and maize ogi porridges were identified as the two most popular porridges for food fortification using *Moringa oleifera* leaf powder and/or *Adansonia digitata* fruit pulp in Benin (Affonfere, 2018), where MNDs are still affecting many children aged 6–59 months. Among these MNDs, iron deficiency (ID) is the commonest, which remains a major public health burden among children (Akpovwa et al., 2020; Kassebaum et al., 2014; Muriuki et al., 2020). More specifically, in Benin, 72% of under 5‐year‐old children have anemia (EDS, 2018), possibly due to insufficient iron intake or sufficient intake combined with impaired absorption due to inflammation and infections such as malaria (Baye et al., 2014; Muriuki et al., 2020). Iron deficiency anemia (IDA) is known to be the most common form of anemia and is estimated to be the cause of up to 50% of anemia cases (WHO, 2008). To overcome this issue, many strategies such as infant and child care feeding practices, control of parasitic infections, food fortification, food diversification, nutritional supplementation, and the use of complementary food supplements were proposed (Adetola et al., 2019; Cardoso et al., 2019; Chadare et al., 2019; Das et al., 2013; Nestel et al., 2003; Tripp et al., 2011).

Complementary food supplements include crushable micronutrient tablets and micronutrient sprinkles that are added to food just before feeding (Nestel et al., 2003). Such complementary food supplements do not require any preparation or addition of water before ingestion and can be stored for long periods without refrigeration, allow individual packaging, and can therefore be used effectively in situations with nonoptimal hygiene conditions (Dibari et al., 2012). Moreover, they can be a good solution to deliver micronutrients to children whose parents cannot afford commercial fortified foods. In that framework, the use of low‐cost, locally available, and nutritious ingredients to formulate complementary food supplements has been recommended (Kunyanga et al., 2012). Chadare et al. (2017) identified and characterized local food ingredients available in the eight agro‐ecological zones of Benin for feeding children aged 6–23 months. Among these food ingredients, *Cochlospermum tinctorium* root powder*, Adansonia digitata* fruit pulp, and *Moringa oleifera* leaf powder are key in terms of their nutritional potential. Specifically, *Cochlospermum tinctorium* root powder was found to contain up to 70.1 mg/100 g dw of iron (Chadare et al., 2017). Its sauce is known to be consumed by under five‐year‐old children in the Sudanian zone of Benin (Affonfere, 2018). *Adansonia digitata* fruit pulp is reported as a vitamin C provider of up to 389 mg/100 g dw (Chadare et al., 2009), which is known to improve mineral bioaccessibility (Gabaza et al., 2018; Van der Merwe et al., 2019). *Adansonia digitata* fruit pulp is also used as a food ingredient by local populations to enrich porridges for under 5‐year‐old children (Affonfere, 2018; Chadare et al., 2008), whereas the leaves of *Moringa oleifera* are used to combat iron deficiencies among children and infants (Shija et al., 2019; Zongo et al., 2013). Furthermore, *Moringa oleifera* leaf powder is recommended for food fortification because of its nutritional potential (Abioye & Aka, 2015; Boateng et al., 2019; Bolarinwa et al., 2019; Karim et al., 2013, 2015). It contains substantial amounts of pro‐vitamin A (624.4 µg retinol equivalent/100 g dw) and vitamin C (65.9 mg/100 g dw), protein (24.3 g/100 g dw), calcium (1,443.9 mg/100 g dw), magnesium (176.7 mg/100 g dw), and iron up to 53.7 mg/100 g dw (Hekmat et al., 2015; Kayalto et al., 2013). Nevertheless, its acceptability is limited when mixed with foods, such as porridges, due to its green color (Karim et al., 2013; Salem et al., 2013). Therefore, it is recommended to combine *Moringa oleifera* leaf powder with other food ingredients to suppress its herbal smell and intense green color (Oyeyinka & Oyeyinka, 2016). Consequently, the present study aimed at formulating an affordable and sensory acceptable complementary food supplement using *Moringa oleifera* leaf powder and other local food ingredients to increase iron intake among children aged 6–59 months in Benin.

## 2 METHODOLOGY

### 2.1 Complementary food supplement ingredients

The local food ingredients to formulate the complementary food supplement were *Moringa oleifera* leaf powder, *Cochlospermum tinctorium* root powder, and *Adansonia digitata* fruit pulp. These were chosen for their availability, nutritional composition, and use as food ingredients (Affonfere, 2018; Chadare et al., ,,^2009^, ^2017^; Houndji et al., 2013; Kayalto et al., 2013). The average costs of these ingredients are 100 FCFA/100 g for *Adansonia digitata* fruit pulp (data from Angel's Floor society in Benin), 40 Fcfa/100 g for *Cochlospermum tinctorium* root powder (data from the local population of Tanguiéta, Benin), and 600 FCFA/100 g for *Moringa oleifera* leaf powder (data from the Hunger Project, Benin). *Moringa oleifera* leaf powder and *Adansonia digitata* fruit pulp were purchased, respectively, at the Hunger Project and Angel's Floor society, two reference centers that produce these ingredients in Benin. *Cochlospermum tinctorium* root powder was collected among the local population of Tanguiéta (Northern Benin), where the species naturally occurs (Akoègninou et al., 2006).

The selected food ingredients and the complementary food supplement were analyzed (in duplicate) for their dry matter, ash, iron (Fe), calcium (Ca), zinc (Zn), magnesium (Mg), phosphorus (P), copper (Cu), sodium (N), manganese (Mn), vitamin C, and total phenolic compounds. Pro‐vitamin A, phytate, and tannin contents of the selected food ingredients were collected from the literature.

#### 2.1.1 Dry matter and ash determination

Dry matter content was assessed by the thermo‐gravimetric method according to AOAC (1993). Samples (5 g) for determining dry matter (Equation 1) were weighted in a crucible and dried in an oven at 105°C for 72 hr. Ash content was determined by dry ash in a furnace at 550°C for 8 h (Equation 2).

(1) $$\text{Dry}\;\text{matter}\;\text{content =}\;\frac{P2‐P1}{\text{Pe}}*100,$$

where *P*1: crucible weight, *P*2: weight (sample +crucible) after drying in the oven, and Pe: weight of sample (5 g)

(2) $$\text{Ash}\;\text{content =}\;\frac{\text{Pf ‐ Pi}}{\text{Pe}}*100,$$

where Pi: porcelain weight, Pf: weight (sample +porcelain) after drying in the furnace, and Pe: weight of the sample (5 g).

#### 2.1.2 Mineral determination

Fe, Ca, Zn, P, Cu, Mg, Na, and Mn contents were determined using atomic absorption spectrometry according to the method described by Pinta (1975). A 5 g sample was placed in a previously weighed porcelain crucible and placed in a muffle furnace at 550ºC for 8 hr. Ash was collected and dissolved in 5 ml of 20% concentrated chloric acid. The content was filtered through Whatman's ashless filter paper, and the volume was brought to 50 ml with bidistilled water. From the solution, Fe, Ca, Zn, P, Cu, Mg, Na, and Mn contents were determined using a flame atomic absorption spectrophotometer (Analyst 200 Perkin Elmer). A standard stock solution of each mineral was prepared in parallel by appropriate dilution.

#### 2.1.3 Vitamin C determination

The vitamin C contents of the selected food ingredients were determined using the 2,6‐dichlorophenolindophenol titrimetric method according to International Standard ISO 6557/2 (1984). The samples were thoroughly mixed with the distilled water, and their filtrates were collected. The filtrate solution (5 ml) was added to oxalic acid (2% v/v) and titrated with freshly prepared 2,6‐dichlorophenolindophenol until obtaining a salmon‐pink coloration that persisted for at least 5 s.

#### 2.1.4 Total phenolic compound determination

Total phenolic compounds were determined according to Singleton and Rossi (1965) as described by Kayodé et al. (2007). Total phenolic compounds were extracted from 50 mg of sample in 1.5 ml of HCl/methanol (1% v/v) for 1 hr under continuous stirring at room temperature. The mixture was centrifuged at 5,000 g for 10 min, after which the supernatant was removed. The pellet was re‐extracted, and the supernatants were pooled. The extracts (300 µl) were mixed with 4.2 ml of distilled water, 0.75 ml of Folin–Ciocalteu's reagent (Merck, Germany), and 0.75 ml of sodium carbonate solution (20% w/v). After incubation for 30 min, the optical density was measured at 760 nm using a spectrophotometer (JENWAY 6850 UV Vis). Blanks were always freshly prepared, in which Folin–Ciocalteu's reagent was replaced by water to correct for interfering compounds. Gallic acid (New Jersey, USA) was used as standard, and the results were expressed as gallic acid equivalent per 100 g dry weight of samples.

### 2.2 Formulation of the complementary food supplement

The complementary food supplement formulation was done by the linear programming functionality of Excel Microsoft (Solver‐Simplex PL) according to Chakeredza et al. (2008). Formulation objectives were set to minimize the cost of the complementary food supplement and to assure that ten grams would cover 25% of the iron daily estimated average requirements for children aged 6 to 12 months, the age‐group with the highest daily estimated average requirements for iron (Ross et al., 2011). The target of a contribution of 25% was chosen as children also consume other sources of iron and to guarantee the acceptability of the complementary food supplement. Next, a constraint was set up for the amount of *Moringa oleifera* leaf powder, based on its acceptability rate in porridges (Abioye & Aka, 2015) and the daily amount of maize porridge consumed by under 5‐year‐old children (Affonfere, 2018). A lower limit of 15% was used for each food ingredient for its contribution to the formula.

### 2.3 Determination of the contribution of the complementary food supplement to the estimated average requirements

The contribution of 10 g (dry weight) of the complementary food supplement to the Fe, Zn, Ca, P, Cu, Mg, and the vitamin C estimated average requirements was assessed (Equation 3). The estimated average requirements proposed by the Food and Nutrition Board/Institute of Medicine (Ross et al., 2011) were used. For Mn, there is no consensus regarding the safe and adequate levels of this nutrient for various age‐groups (Avila et al., 2013).

(3) $$\text{Cover}\;\text{rate}\;\left(\%\right)=\frac{\text{Minerals}\;\text{or}\;\text{vitamin content in 10 g dry base CFS*100}\;\;}{\text{Minerals or vitamin EARs}}$$

### 2.4 Determination of minerals to phytate molar ratios and vitamin C to iron molar ratio

The inhibitory effect of dietary phytate on the bioavailability of minerals from the diet can be estimated through phytate minerals molar ratios (Al Hasan et al., 2016). In the present study, the phytate contents of selected ingredients were collected from the literature and the phytate content of the complementary food supplement was theoretically calculated accordingly. The moles of phytate and minerals were determined by dividing the weights (contents) of phytate and minerals with their atomic weights (phytate: 660 g/mol; Fe: 56 g/mol; Zn: 65 g/mol; Ca: 40 g/mol). The molar ratios between phytate and minerals were obtained after dividing the mole of phytate with the mole of minerals (Norhaizan & Nor Faizadatul Ain, 2009). The same approach was used for the vitamin C: iron molar ratio. The atomic weight used for vitamin C was 176.12 g/mol.

### 2.5 Acceptability of the complementary food supplement

The acceptability of the complementary food supplement was assessed by adding the supplement to porridges made from sorghum and maize ogi (Affonfere, 2018). The fortification rates for the acceptability test were defined based on the quantities of sorghum and maize porridges daily consumed by under five‐year‐old children according to the aforementioned author and the 10 g of complementary food supplement to be consumed daily by the children. Thus, 16% and 15% fortification rates (dry basis) were tested for sorghum and maize ogi porridges, respectively, using five (5) hedonic scale points. The test was performed in Natitingou and Materi (Northern Benin), where populations are accustomed to the consumption of sorghum and maize ogi porridges fortified with *Moringa oleifera* leaf powder and/or *Adansonia digitata* fruit pulp. Eighty (80) mothers and eighty (80) children aged 6–59 months participated voluntarily, and all mothers signed a consent form. The fortified porridges were evaluated at 45°C, corresponding to the common consumption temperature for porridge by infants (Mouquet & Treche, 2006). Fifty grams of each fortified porridge was served to the panelist in a white cup, and clean water was provided to rinse the mouth between evaluations. During the consumption of the fortified porridges, the facial expressions of the children were recorded by the investigators using facial hedonic scales of five (05) levels (0: very bad, 1: bad, 2: maybe good or bad, 3: good, and 4: very good) as described by Guinard (2001). These nonverbal cues have previously been recommended (Guinard, 2001) and used in similar studies (Mithamo et al., 2020; Ngoma et al., 2018). The use of nonverbal cues is the practice of choice in the baby food industry (Kevin, 1995). Mothers evaluated the fortified porridges using the sensory attributes under evaluation after their training on how to use a hedonic scale of five (05) levels. The attributes were acidity, color, smell, consistency, and overall acceptability.

### 2.6 The physicochemical characteristics of enriched and unenriched porridges

The physicochemical characteristics of enriched and unenriched maize and sorghum ogi porridges were determined in triplicate. These physicochemical characteristics were pH, titratable acidity, Brix value, lightness L*, redness a*, yellowness b*, total color difference ΔE, and consistency. Sample pH was measured with a pH meter (Eutech Instruments pH 510) according to ISO1842 (EAS, 2000). The titratable acidity was determined using 0.1 N of NaOH as titration solution according to the modified norm AACC 02‐31.01. The Brix values were determined using an ATAGO digital refractometer according to ISO 2173:2003. The lightness L*, redness a*, yellowness b*, and total color difference ΔE were measured according to the standards of the International Committee of Lighting (CIELAB, 1976), using a Chromameter CR‐410 (Konica Minolta optics, INC, Osaka, Japan). The device was calibrated with a standard white plate using the D65 illuminant (*Y* = 86.1; *x* = 0.3194; *y* = 0.3369). The consistency of the enriched and unenriched porridges was determined by measurement in a Bostwick consistometer. The Bostwick flow rate was expressed in mm/30 s as described by El Tou et al. (2002).

### 2.7 Statistical analyses

Results were expressed as mean ± standard deviation per 100 g sample (dry weight). One‐way analysis of variance (ANOVA) with Minitab version 18 was performed for physical characteristics to check the effect of the complementary food supplement on the maize and sorghum ogi porridges. A significant difference was accepted at *p* < .05. In case of a difference, the Tukey test was performed to separate means, with a confidence level for significant differences at *p* < .05. A significant difference between each sensory attribute scale was checked using the multiple proportion chi‐square test. A correlation test (Spearman) was also performed to assess relations between sensory attributes and the overall liking of the enriched porridges.

## 3 RESULTS

### 3.1 Composition of the complementary food supplement

Table 1 presents the ingredients in g per 100 g of the complementary food supplement. The results show that *Adansonia digitata* fruit pulp is the major ingredient (65%), followed by the *Cochlospermum tinctorium* root powder (20%) and *Moringa oleifera* leaf powder (15%). The estimated cost of 100 g (wet basis) of the complementary food supplement was FCFA 195.9, which is approximately $ 0.33.

**TABLE 1:** Composition of the complementary food supplement and its estimated cost

Ingredients	Quantity (dry basis)	Quantity (wet basis)	Quantity for 100 g wet basis	Cost (FCFA) for 100 g (wet basis)	Cost ($) for 100 g (wet basis)^a^
*Adansonia digitata* fruit pulp	63	73.5	64.8	73.5	0.12
*Moringa oleifera* leaf powder	15	16.5	14.6	99.0	0.17
*Cochlospermum tinctorium* root powder	22	23.3	20.6	23.3	0.04
Complementary food supplement	100	113.4	100.0	195.9	0.33

^a^ The exchange rate considered is 1 $ = 583.50 Franc CFA.

### 3.2 Nutritional composition of the food ingredients and complementary food supplement

#### 3.2.1 Minerals

*Moringa oleifera* leaf powder was found to be rich in minerals, followed by *Cochlospermum tinctorium* root powder and *Adansonia digitata* fruit pulp (Table 2). The mineral content of *Adansonia digitata* fruit pulp, expressed as mg/100 g dw, was 9.9 ± 0.1 for iron, 0.9 ± 0.1 for zinc, and 402.2 ± 3.4 for calcium. The mineral content of *Moringa oleifera* leaf powder, in mg/100 g dw, was 34.1 ± 2.2 for iron, 1.9 ± 0.0 for zinc, and 2054.9 ± 11.5 for calcium. The iron, zinc, and calcium content of *Cochlospermum tinctorium* root powder, again in mg/100 g dw, were 26.8 ± 2.7, 0.9 ± 0.0, and 1,061.3 ± 11.5, respectively. The formulated complementary food supplement contained, in mg/100 g dw, 17.4** ± **1.1 of iron, 1.2 ± 0.1 of zinc, and 830.0 ± 0.2 of calcium. Except for copper, significant differences were found between the mineral content of the ingredients and the developed complementary food supplement (Table 2).

**TABLE 2:** Dry matter (%), ash (%), and mineral (mg/100 g dw) contents of the selected food ingredients and the complementary food supplement

Food ingredients and complementary food supplement	Dry matter	Ash	Iron	Zinc	Calcium	Magnesium	Manganese	Phosphorus	Copper	Sodium
*Adansonia digitata* fruit pulp	85.7 ± 0.2^a^	5.1 ± 0.1^a^	9.9 ± 0.1^a^	0.9 ± 0.1^a^	402.2 ± 3.4^a^	153.0 ± 1.6^a^	0.4 ± 0.0^a^	68.9 ± 15.5^a^	0.4 ± 0.1^a^	16.7 ± 0.0^a^
*Moringa oleifera* leaf powder	90.9 ± 0.2^b^	8.5 ± 0.2^b^	34.1 ± 2.2^b^	1.9 ± 0.0^b^	2054.9 ± 11.5^b^	616.7 ± 8.9^b^	10.1 ± 0.0^b^	271.9 ± 4.1^b^	0.6 ± 0.2^a^	74.3 ± 1.3^b^
*Cochlospermum* *tinctorium* root powder	94.1 ± 0.4^c^	7.0 ± 0.0^c^	26.8 ± 2.7^c^	0.9 ± 0.0^a^	1,061.3 ± 37.4^c^	369.4 ± 1.9^c^	2.5 ± 0.0^c^	91.9 ± 2.1^c^	nd	12.4 ± 1.0^c^
Complementary food supplement	86.3 ± 0.4^a^	5.8 ± 0.0^d^	17.4 ± 1.1^d^	1.2 ± 0.1^c^	830.0 ± 0.2^d^	292.0 ± 4.1^d^	2.5 ± 0.0^c^	107.9 ± 6.6^c^	0.3 ± 0.2^a^	17.9 ± 3.2^a^

Abbreviations: Nd, not detected.

#### 3.2.2 Vitamins and antinutritional factors

The contents of vitamin A and vitamin C and the antinutritional factors of *Adansonia* *digitata* fruit pulp, *Moringa oleifera* leaf powder, *Cochlospermum tinctorium* root powder, and the complementary food supplement are presented in Table 3. *Adansonia digitata* fruit pulp had 372.7 ± 12.2 mg/100 g dw of vitamin C and 2,128.2 ± 44.5 mg eq AG/100 g dw of total phenols. *Moringa oleifera* leaf powder and *Cochlospermum tinctorium* root powder had, respectively, 24.6 ± 1.4 mg/100 g dw and 23.4 ± 1.3 mg/g dw of vitamin C and 2,256.7 ± 259.0 mg eq AG/100 g dw and 2,694.6 ± 29.8 mg eq AG/100 g dw of total phenols. The complementary food supplement contained 243.6 ± 11.1 mg/100 g dw of vitamin C and 2,221.9 ± 83.6 mg eq AG/100 g dw of total phenols. The phytate/iron molar ratio of the complementary food supplement was 2.03:1, while the phytate/calcium and phytate/zinc molar ratios were 0.03:1 and 34.99:1, respectively. The vitamin C: iron molar ratio was 4.5:1.

**TABLE 3:** Vitamins and antinutritional factors of the selected food ingredients and complementary food supplement

Food ingredients and complementary food supplement	Vitamin C (mg/100 g dw)	Vitamin A (mg/100 g dw)^*^	Phenol (mg eq AG/100 g dw)	Phytate (mg/100 g dw)^*^	Tanin (mg/100 g dw)^*^
*Adansonia digitata* fruit pulp	372.7 ± 12.2^a^	NA	2,128.2 ± 44.5^a^	287.5 ± 47.4	8,353.0 ± 201.0
*Moringa oleifera* leaf powder	24.6 ± 1.4^b^	19.7 ± 1.0	2,256.7 ± 259.0^ab^	829.0 ± 23.0	37.0 ± 1.0
*Cochlospermum* *tinctorium* root powder	23.4 ± 1.3^b^	NA	2,694.6 ± 29.8^b^	500.0 ± 200	NA
Complementary food supplement	243.6 ± 11.1^c^	2.96^**^	2,221.9 ± 83.6^ab^	415.38^**^	5,271.60^**^

^*^ Data from literature (Adetola et al., 2019; Leone et al., 2015; Mumtaz & Fatima, 2017; Van der Merwe et al., 2019).

^**^ Theoretic values.

### 3.3 Contribution of the 10 g complementary food supplement to the estimated average requirements

The study findings indicate that ten (10) grams of complementary food supplement covers 25% of the iron estimated average requirements for 6‐ to 12‐month‐old children and 58% and 42% of the iron estimated average requirements for 12‐ to 36‐month‐old children and 36‐ to 59‐month‐old children, respectively (Table 4). For zinc, the daily consumption of 10 g complementary food supplement would cover 5% of zinc estimated average requirements for children under 36 months old and less than 3% for 36‐ to 59‐month‐old children. The consumption of 10 g complementary food supplement per day would also cover 17% and 10% of the calcium estimated average requirements for 12‐ to 36‐month‐old and 36‐ to 59‐month‐old children, respectively. For vitamin C, the indicated consumption level of the complementary food supplement would contribute the total vitamin C estimated average requirements for 12‐ to 59‐month‐old children and 187% and 111% for 12‐ to 36‐month‐old and 36‐ to 59‐month‐old children, respectively.

**TABLE 4:** Contribution (%) of the 10 g complementary food supplement to the minerals and vitamin C estimated average requirements

Age‐groups	Fe		Zn		Ca		Mg		P		Cu		Vitamin C	
	EARs (mg/d)	Cover rate (%)	EARs (mg/d)	Cover rate (%)	EARs (g/d)	Cover rate (%)	EARs (mg/d)	Cover rate (%)	EARs (mg/d)	Cover rate (%)	EARs (µg/d)	Cover rate (%)	EARs (mg/d)	Cover rate (%)
6–12 m	6.9	25.2	2.5	4.7	NA	NA	NA	NA	NA	NA	NA	NA	NA	NA
12–36 m	3	57.9	2.5	4.7	0.5	16.5	65	44.9	380	2.8	260	12.9	13	187.3
36–59 m	4.1	42.4	4	2.9	0.8	10.3	110	26.5	405	2.7	340	9.9	22	110.7

Abbreviations: d, day; EARs, estimated average requirements; m, month; NA, not available.

### 3.4 Acceptability of the complementary food supplement

**FIGURE 1:**
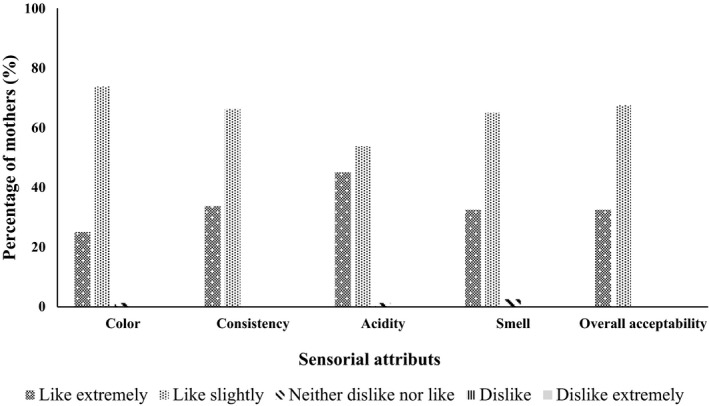
Acceptability level of enriched sorghum ogi porridge at 16% of fortification rate by mothers (*n* = 80)

**FIGURE 2:**
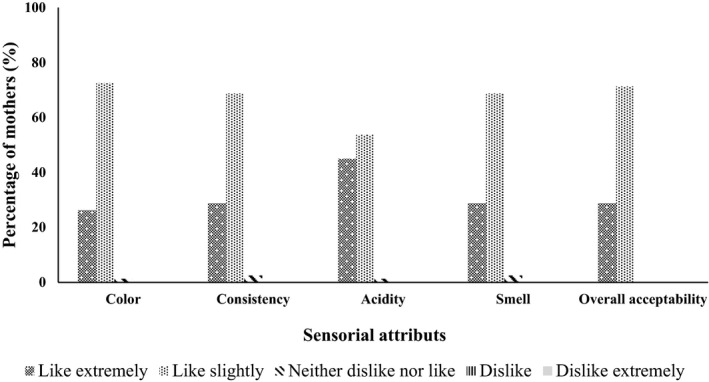
Acceptability level of enriched maize ogi porridge at 15% of fortification rate by mothers (*n* = 80)

Seventy‐five percent (75%) of enrolled mothers were 18 to 30 years old and twenty‐five percent (25%) more than 30 years old. Thirty percent (30%) of children were 6–12 months old, 45% were between 13 and 24 months old, and 25% were between 24 and 59 months old. Sixty‐four percent (64%) of the children extremely liked the enriched sorghum ogi porridge, while 21% of them like it slightly, 14% were indifferent, and 1% disliked it. Regarding enriched maize ogi porridge, 66% of children liked the porridge extremely, 21% liked it slightly, and 13% were indifferent. Mothers extremely and slightly liked the enriched sorghum ogi porridge color (25% and 74%, respectively), consistency (34% and 66%), acidity (45% and 54%), and smell (33% and 65%). The overall acceptability showed that 33% of the mothers extremely liked the enriched sorghum ogi porridge, while 67% liked it slightly (Figure 1). For enriched maize ogi porridge, more than 25% of the mothers extremely liked the enriched maize ogi porridge regarding color, consistency, acidity, and smell. Overall, 29% of mothers extremely liked the enriched maize ogi porridge and 71% liked it slightly (Figure 2). For each of these attributes, the proportion of the acceptability scales were significantly different (*p* < .0001). The overall liking of enriched sorghum ogi porridge correlated most with the consistency (*r* = 0.859, *p* < .0001), followed by the acidity (*r* = 0.761, *p* < .0001). In the case of enriched maize ogi porridge, the consistency and smell correlated most with the overall liking (*r* = 0.740, *p* < .0001), followed by the acidity (*r* = 0.697; *p* < .0001). Color correlated least with the overall liking of the enriched porridges.

### 3.5 Physicochemical characteristics of enriched and unenriched ogi porridges

Table 5 shows the physicochemical characteristics of enriched maize and sorghum ogi porridges with the complementary food supplement and unenriched maize and sorghum ogi porridges.

**TABLE 5:** Physicochemical characteristics of unenriched and enriched maize and sorghum ogi porridge with the complementary food supplement

Food matrices	pH	Titratable acidity (% lactic acid per 100 grams dw)	Degrees Brix (%)	L	a	b	ΔE	Bostwick flow rate (mm/30 s)
Sorghum ogi porridge	4.21 ± 0.03^a^	0.11 ± 0.00^a^	5.2 ± 0.1^a^	60.3 ± 0.8^a^	11.0 ± 0.2^a^	15.8 ± 0.2^a^	37.8 ± 0.6^a^	˃23
Sorghum ogi porridge +complementary food supplement	3.93 ± 0.02^b^	0.25 ± 0.00^b^	6.0 ± 0.1^b^	55.6 ± 0.3^b^	6.2 ± 0.0^b^	21.4 ± 0.1^a^	42.7 ± 0.2^a^	22.6 ± 0.0
Maize ogi porridge	3.86 ± 0.02^*^	0.12 ± 0.00^*^	5.9 ± 0.2^*^	80.7 ± 0.2^*^	−1.1 ± 0.0^*^	7.1 ± 0.1^*^	14.0 ± 0.1^*^	8.4 ± 0.4^*^
Maize ogi porridge +complementary food supplement	3.83 ± 0.04^*^	0.29 ± 0.00^**^	6.3 ± 0.2^**^	62.2 ± 0.4^**^	1.8 ± 0.1^**^	26.0 ± 0.0^**^	39.0 ± 0.3^**^	6.9 ± 0.1^**^

Note

For each physicochemical parameter and each enriched and unenriched porridge, means (±standard deviation) with the same letter or the same number of star are not significantly different (*p* > .05).

#### 3.5.1 Unenriched and enriched sorghum ogi porridge

After enrichment with the complementary food supplement, the pH of sorghum ogi porridge decreased significantly from 4.2 ± 0.03 to 3.93 ± 0.02. The consistency of this porridge was 22.6 mm/30 s, while the unenriched sorghum ogi porridge had a consistency above 23 mm/30 s. The degrees Brix, total color difference, and titratable acidity significantly increased from 5.2 ± 0.1% to 6.0 ± 0.1%, 37.9 ± 0.6 to 42.7 ± 0.2, and 0.11 ± 0.00 to 0.25 ± 0.00% lactic acid per 100 gram dw, respectively.

#### 3.5.2 Unenriched and enriched maize ogi porridge

The pH of maize ogi porridge decreased but not significantly from 3.86 ± 0.02 to 3.83 ± 0.04 after the enrichment with the complementary food supplement. In contrast, the degrees Brix and total color difference increased significantly after the enrichment from 5.9 ± 0.2 to 6.3 ± 0.2% and from 14.0 ± 0.1 to 39.0 ± 0.3, respectively. The Bostwick flow rate (mm/30 s) after the enrichment of maize ogi porridge decreased significantly from 8.4 ± 0.4 to 6.9 ± 0.1.

## 4 DISCUSSION

### 4.1 Nutritional potential of the selected food ingredients

The iron contents of *Adansonia digitata* fruit pulp and *Moringa oleifera* leaf powder in this study were in line with the values in the literature (Adetola et al. 2019; Chadare et al. 2009; Van der Merwe et al. 2019). To the best of our knowledge, only one study conducted by Chadare et al. (2017) investigated the mineral content of *Cochlospermum tinctorium* root powder. The authors found a value of 70.1 mg/100 g dw for iron, which is higher than the value in this study (26.8 ± 2.7 mg/100 g dw). This discrepancy could be explained by the genetic background, the environmental conditions, the analytical methods, and the provenance of the sample (Chadare et al., 2009; Kayalto et al., 2013; Moyo et al., 2011; Muthai et al., 2017; Stadlmayr et al., 2013). Nevertheless, the iron content of *Cochlospermum tinctorium* root powder in this study still makes it suitable to be promoted as a local food fortificant to address the iron deficiency issue as suggested by Affonfere (2018). *Cochlospermum tinctorium* root powder can be successfully used in combination with *Moringa oleifera* leaf powder for food fortification to improve the iron status of children when *Moringa oleifera* leaf powder is not available and/or to mitigate its high cost, as *Moringa oleifera* leaf powder is fifteen (15) times more expensive than *Cochlospermum tinctorium* root powder. The vitamin C content of *Adansonia digitata* fruit (372.7 ± 12.2 mg/100 g dw) found in this study can contribute to achieving the vitamin C requirement for children under five years old. This is also important to improve the bioaccessibility of minerals, especially iron. The high calcium content of *Moringa oleifera* leaf powder and *Cochlospermum tinctorium* root powder is also beneficial to children. Calcium is important to calcification and dentition for children (Soetan et al., 2010).

### 4.2 Affordability and nutritional benefits of the complementary food supplement: implication for alleviation of iron deficiency

The food ingredients of the complementary food supplement were locally available, and the processes to obtain these ingredients do not require any specific equipment. Local populations can therefore easily formulate the complementary food supplement themselves. The complementary food supplement is also affordable for rural populations (0.33 $/100 g) with limited incomes (IFPRI, 2019). Our research findings showed that daily consumption of 10 g (dry basis) of this complementary food supplement will provide 1.7 mg of iron, 0.1 mg of zinc, 83 mg of calcium, and 24.3 mg of vitamin C. This daily consumption covers 25% of iron estimated average requirements for 6‐ to 12‐month‐old children and 58% and 42% of iron estimated average requirements for 12‐ to 36‐month‐old and 36‐ to 59‐month‐old children, respectively. Based on the aforementioned data, the formulated complementary food supplement can be used to alleviate iron deficiency among under five‐year‐old children.

The inhibitory effect of phytic acid on mineral absorption, especially calcium, iron, and zinc, is well known (Al Hasan et al., 2016; Hurrell & Egli, 2010), and phytate–mineral molar ratios were suggested to assess this effect (Al Hasan et al., 2016). The phytate/iron molar ratio of the complementary food supplement is 2.03:1, which is lower than the critical value (10–14) above which phytate is believed to strongly impair iron absorption (Saha et al., 1994). Accordingly, good iron bioavailability may be expected from the complementary food supplement. Furthermore, vitamin C is known to be a good promoter of iron absorption (Zimmermann et al., 2005). Due to the considerable vitamin C content of the complementary food supplement (vitamin C: iron molar ratio of 4.5:1), this will improve the iron bioavailability. Zimmermann et al. (2005) reported that a vitamin C: iron molar ratio ≥4:1 is needed for optimal iron absorption in food with high phytate content. The phytate/calcium molar ratio of the complementary food supplement is 0.03:1, which is lower than the critical upper value of 0.24 (Morris & Ellis, 1985), while the phytate/zinc molar ratio is 34.99:1, which higher than the critical upper value of 10–15 (Morris & Ellis, 1985; Oberleas & Harland, 1981; Turnlund et al., 1984). These molar ratios suggest that the phytate content of the complementary food supplement might impair zinc absorption. Apart from phytate, other antinutritional factors such as phenolic compounds could also impair mineral absorption. Hence, assessment of mineral bioavailability is recommended for the complementary food supplement and enriched porridges.

### 4.3 Acceptability of the complementary food supplement

The maize and sorghum ogi porridges enriched at 15% and 16% dw, respectively, were well accepted by the mothers and children. A similar trend was observed by Affonfere (2018) when the author found that the sorghum ogi porridge fortified with both *Moringa oleifera* leaf powder and *Adansonia digitata* fruit pulp was accepted at up to 17% dw fortification. Maize porridge, sorghum, or millet porridges fortified with *Moringa oleifera* leaf powder were found to be accepted up to 10% dw, above which the green color of *Moringa oleifera* leaf powder made the fortified foods unacceptable (Abioye & Aka, 2015; Olorode et al., 2013). *Adansonia digitata* fruit pulp in combination with *Moringa oleifera* leaf powder seems to have the potential to increase the acceptable level of fortification, possibly due to the acidulating effect of *Adansonia digitata* fruit pulp. Several authors demonstrated good acceptability of *Adansonia digitata* fruit pulp at high fortification levels in various foods: 20% for ogi (Adejuyitan et al., 2012), 40% for yoghurt (Abdullahi et al., 2014), and 20% for cooked rice (Mounjouenpou et al., 2018).

The overall acceptability underlined the generally positive responses from mothers (100% of the mothers liked both enriched porridges). Mothers stated that the maize and sorghum ogi porridges enriched with the complementary food supplement could be consumed without sugar. Indeed, after the enrichment, the porridges’ degrees Brix increased significantly. This increase is attributed to the refreshing taste and natural sugar content of *Adansonia digitata* fruit pulp (De Caluwé et al., 2010; Rahul et al., 2015). De Caluwé et al. (2010) confirmed that *Adansonia digitata* fruit pulp is acidulous and sweet. The aforementioned authors added that this acidulous taste is due to the presence of organic acids, including citric, tartaric, malic, succinic, and ascorbic acids, while its sweetness is provided by the fructose, saccharose, and glucose in *Adansonia digitata* fruit pulp. Although the complementary food supplement changed the color of the porridges significantly, the mothers appreciated the enriched porridges. This is because the color correlated less with the overall liking than the smell, consistency, and acidic taste. According to Boateng et al. (2019), especially taste is an important influential factor in a person's selection of a particular food.

Regarding the children, the analyses of their nonverbal cues showed that most of them (87% for sorghum and 85% for maize porridges) liked the enriched porridges. The positive responses from children and their mothers regarding the acceptability of the enriched porridges will support the adoption of the developed product as acceptability is key to predict the intent to use a product (Barcenilla & Bastien, 2009; Février, 2011). Adoption here refers to an individual (or collective) decision to accept and use a product (Alexandre et al., 2018). Additionally, the low cost of the complementary food supplement, as well as the physical availability of the food ingredients, will contribute to the adoption of the developed product. Promotion of complementary food supplement will enhance iron intake among children. It is recommended that nutrition education and counseling be provided to mothers and caregivers of under five‐year‐old children to ensure proper nutrition within the target group. Finally, further research could investigate the acceptability of the complementary food supplement in other food matrices at varying fortification rates.

## 5 CONCLUSION

This study demonstrated the potential of local food ingredients in the formulation of a sensory acceptable complementary food supplement, which can be used by local populations to enhance their iron intake. The 10 g dry weight corresponding to 11.34 g wet weight daily consumption of the complementary food supplement covers 25 to 57% of iron estimated average requirements for children from 6 to 59 months old.

## CONFLICTS OF INTEREST

The authors declare that they do not have any conflict of interest.

## AUTHOR CONTRIBUTIONS

**Marius Affonfere:** Conceptualization (equal); Data curation (equal); Investigation (equal); Methodology (equal); Software (equal); Writing‐original draft (lead); Writing‐review & editing (equal). **Flora Josiane Chadare:** Conceptualization (lead); Methodology (lead); Project administration (lead); Supervision (lead); Validation (lead); Writing‐review & editing (equal). Finagnon **Toyi Kévin Fassinou:** Investigation (equal); Methodology (supporting); Software (equal); Writing‐review & editing (supporting). **Elise F. Talsma:** Writing‐review & editing (equal). **Anita Linnemann:** Writing‐review & editing (equal). **Paulin AZOKPOTA:** Writing‐review & editing (equal).

## ETHICAL STATEMENTS

Ethical Review: This study does not involve any human or animal testing.

Informed Consent: Written informed consent of participants was obtained for the acceptability test after receiving complete information about the study in the local language.
